# Cerebral white matter disease and functional decline in older adults from the Northern Manhattan Study: A longitudinal cohort study

**DOI:** 10.1371/journal.pmed.1002529

**Published:** 2018-03-20

**Authors:** Mandip S. Dhamoon, Ying-Kuen Cheung, Yeseon Moon, Janet DeRosa, Ralph Sacco, Mitchell S. V. Elkind, Clinton B. Wright

**Affiliations:** 1 Department of Neurology, Icahn School of Medicine at Mount Sinai, New York, New York, United States of America; 2 Department of Biostatistics, Mailman School of Public Health, Columbia University, New York, New York, United States of America; 3 Department of Neurology, College of Physicians and Surgeons, Mailman School of Public Health, Columbia University, New York, New York, United States of America; 4 Evelyn McKnight Brain Institute, Miller School of Medicine, University of Miami, Miami, Florida, United States of America; 5 Departments of Neurology, Public Health Sciences and Human Genetics, Miller School of Medicine, University of Miami, Miami, Florida, United States of America; 6 Department of Epidemiology, Mailman School of Public Health, Columbia University, New York, New York, United States of America; 7 National Institutes of Health, Bethesda, Maryland, United States of America; National University of Singapore, SINGAPORE

## Abstract

**Background:**

Cerebral white matter hyperintensities (WMHs) on MRI are common and associated with vascular and functional outcomes. However, the relationship between WMHs and longitudinal trajectories of functional status is not well characterized. We hypothesized that whole brain WMHs are associated with functional decline independently of intervening clinical vascular events and other vascular risk factors.

**Methods and findings:**

In the Northern Manhattan Study (NOMAS), a population-based racially/ethnically diverse prospective cohort study, 1,290 stroke-free individuals underwent brain MRI and were followed afterwards for a mean 7.3 years with annual functional assessments using the Barthel index (BI) (range 0–100) and vascular event surveillance. Whole brain white matter hyperintensity volume (WMHV) (as percentage of total cranial volume [TCV]) was standardized and treated continuously. Generalized estimating equation (GEE) models tested associations between whole brain WMHV and baseline BI and change in BI, adjusting for sociodemographic, vascular, and cognitive risk factors, as well as stroke and myocardial infarction (MI) occurring during follow-up. Mean age was 70.6 (standard deviation [SD] 9.0) years, 40% of participants were male, 66% Hispanic; mean whole brain WMHV was 0.68% (SD 0.84). In fully adjusted models, annual functional change was −1.04 BI points (−1.20, −0.88), with −0.74 additional points annually per SD whole brain WMHV increase from the mean (−0.99, −0.49). Whole brain WMHV was not associated with baseline BI, and results were similar for mobility and non-mobility BI domains and among those with baseline BI 95–100. A limitation of the study is the possibility of a healthy survivor bias, which would likely have underestimated the associations we found.

**Conclusions:**

In this large population-based study, greater whole brain WMHV was associated with steeper annual decline in functional status over the long term, independently of risk factors, vascular events, and baseline functional status. Subclinical brain ischemic changes may be an independent marker of long-term functional decline.

## Abstract 2

Using data from the Northern Manhattan Study, Mandip and colleagues examine the association between cerebral white matter disease and functional decline in older adults.

## Author summary

**Why was this study done?:**

- White matter changes on brain MRI are common and associated with vascular and functional outcomes.
- However, we do not know about the relationship between whole brain white matter hyperintensities (WMHs) and functional status over time, even though it is known that functional status is not static over time.
- We hypothesized that WMHs are associated with steeper functional decline, even when adjusting for clinical vascular events and other vascular risk factors.

**What did the researchers do and find?:**

- In the Northern Manhattan Study, a population-based racially/ethnically diverse prospective cohort study, 1,290 stroke-free individuals underwent brain MRI and were followed afterwards for a mean 7.3 years with annual functional assessments using the Barthel index (BI).
- We examined associations between whole brain white matter hyperintensity volume (WMHV) (as percentage of total cranial volume) and baseline BI and change in BI, adjusting for sociodemographic, vascular, and cognitive risk factors and stroke and myocardial infarction (MI) occurring during follow-up.
- We found, in fully adjusted models, that annual functional change was −1.04 BI points (−1.20, −0.88), with −0.74 additional points annually per SD whole brain WMHV increase from the mean (−0.99, −0.49).

**What do these findings mean?:**

- The steeper annual decline in functional status seen with greater whole brain WMHV was seen even among those with no disability at baseline, which emphasizes the “subclinical” nature of these predictors and their strong predictive power on trajectories of functional status.

## Introduction

Functional status reflects performance in activities of daily living (ADLs) and instrumental ADLs, which are outcomes that are of primary importance to elderly patients and funding agencies. Although the determinants of functional status among the elderly have been extensively studied, there is a relative lack of understanding about the influence of subclinical cerebrovascular disease on the course of functional status over years. In particular, there is little data regarding the time course of change in functional trajectories in relation to white matter hyperintensities (WMHs) and the specific aspects of function that are compromised by white matter disease. Also, few studies have accounted for the effects of clinically evident vascular events intervening during follow-up in a reliable manner.

Cerebral white matter lesions likely represent white matter structural damage due to vascular disease. WMHs are most probably caused by traditional vascular risk factors [1], and they have been associated with vascular outcomes, including stroke [2] and mortality [3], as well as cognitive impairment [4] and functional impairment [5–8]. In a prior cross-sectional analysis in the Northern Manhattan Study (NOMAS) [9], higher whole brain white matter hyperintensity volume (WMHV) was associated with poorer episodic memory, processing speed, and semantic memory. In a prior longitudinal analysis in NOMAS, we found that asymmetry of WMHV was independently associated with long-term functional decline [10]. In this study, we hypothesized that whole brain WMHV was independently associated with worse baseline functional status and slope of change over time in those free of stroke at baseline. There are several novel approaches of this analysis. First, we estimated 2 components of functional trajectories: baseline function and change over time. Estimating trajectories may reveal courses of change and specific predictors that are not captured with crude analysis of change over 2 time points. Also, we analyzed the components of function using subdomains of a functional scale.

## Methods

The NOMAS MRI study is a substudy of the NOMAS prospective cohort [11] that began in 2003 and included 1,290 individuals who were: 1) of an age ≥50 years, 2) without contraindications to MRI, 3) without clinical stroke, and 4) able to provide written informed consent. Imaging was performed on a 1.5T MRI system (Philips Medical Systems, Best, Netherlands) and included axial T1, axial T2, and Fluid Attenuated Inversion Recovery sequences. An operator traced dura mater, and non-brain structures were manually removed from images. Using a custom-designed image analysis package, modeling of pixel-intensity histograms for cerebral spinal fluid (CSF) and brain white and gray matter was performed. Semiautomated measurements of pixel distributions were made to identify the optimal pixel-intensity threshold to distinguish CSF from brain matter. Total cranial volume (TCV) constituted the sum of whole brain volume voxels from the T1 segmentation process. Whole brain WMHV was calculated as the sum of voxels ≥3.5 standard deviations (SD) above mean image intensity multiplied by pixel dimensions and section thickness [9]. WMHV was divided by TCV, multiplied by 100 to yield percent TCV, subtracted from the mean, and divided by the SD. Subclinical brain infarcts (SBIs) were lesions >3 mm, distinct from the circle of Willis in the basal ganglia, and of similar intensity as CSF. Columbia University and University of Miami IRBs approved the study. This study is reported as per the Strengthening the Reporting of Observational Studies in Epidemiology (STROBE) guidelines (S1 Text).

### Baseline evaluation

Standardized questions captured the following conditions: hypertension, diabetes mellitus, hypercholesterolemia, cigarette smoking, alcohol use, and cardiac conditions [12]. Baseline examination included comprehensive medical history, physical examination, medical record review, functional status assessed by the Barthel index (BI), quality of life (QOL) assessed by the Spitzer QOL index, cognitive performance measured by the mini-mental state examination (MMSE) [13], and fasting blood samples.

### Follow-up

Participants were followed up with annually via phone to detect death, capture new neurological or cardiac symptoms and events and interval hospitalizations, and measure functional status via the BI. Only 2 subjects were lost to follow-up after their baseline examination, and the average annual contact rate was 99%.

A potential cardiac or neurological event was followed by an in-person assessment to determine whether a vascular outcome occurred. All admissions and discharges of NOMAS study participants to Columbia University Medical Center (CUMC) were additionally screened for possible outcome events. Nearly 70% of vascular events in the cohort lead to hospitalizations at CUMC. Hospital records were reviewed to classify outcomes as previously reported [14]. Stroke included ischemic stroke, intracerebral hemorrhage, and subarachnoid hemorrhage. At least 2 stroke neurologists verified and classified stroke cases. Myocardial infarction (MI) required ≥2 of these criteria: (a) typical angina ischemic cardiac pain; (b) abnormal CK-MB fraction or troponin-I values; (c) ischemic ECG abnormalities. Cardiologists adjudicated MI diagnoses.

### Study outcome

The BI [15,16] measures 10 ADLs and ranges from 0–100, with 100 indicating normal functioning. The 10 ADLs and the range of possible scores for each are as follows: Feeding (0, 5, 10), Bathing (0, 5), Grooming (0, 5), Dressing (0, 5, 10), Bowels (0, 5, 10), Bladder (0, 5, 10), Toilet use (0, 5, 10), Transfers (0, 5, 10, 15), Mobility (0, 5, 10, 15), and Stairs (0, 5, 10). Previous research has demonstrated the reliability of phone BI assessments [17]. The scale may be analyzed as a continuous variable for increased power to detect associations, ability to describe the course of change over time in linear form, and avoidance of potential misclassification due to crude categorization [18–20]. BI measurements from the time of MRI forward were included.

### Covariates

Analytic models were adjusted for: demographic variables (age, sex, race/ethnicity), medical risk factors (body mass index [body weight in kilograms divided by the square of height in meters], hypercholesterolemia [defined by self-report, lipid-lowering therapy use, or fasting total cholesterol level >240 mg/dL], diabetes mellitus [defined by self-report, fasting blood glucose level ≥126 mg/dL, or insulin/oral hypoglycemic use], hypertension [defined as a systolic blood pressure recording ≥140 mmHg or a diastolic blood pressure recording ≥90 mm Hg based on the average of 2 blood pressure measurements or the participant’s self-report of a history of hypertension or antihypertensive use]), smoking (defined as either nonsmoker or smoker within the last year), alcohol use (with moderate alcohol use classified as 1 drink/month to 2 drinks/day), any physical activity (versus none), social variables (marital status, insurance status [classified uninsured/Medicaid versus Medicare/private insurance], number of friends [individuals whom the participant knows well enough to visit in their homes], years living in the community), and cognitive/mood factors (depressed mood, performance on MMSE [analyzed as a continuous variable], and Spitzer QOL index score).

### Statistical analysis

We have followed a prospective analysis plan without alteration (S2 Text). We calculated variable distributions using means and SDs for continuous variables and frequency and percentage for categorical variables.

**Fig 1:**
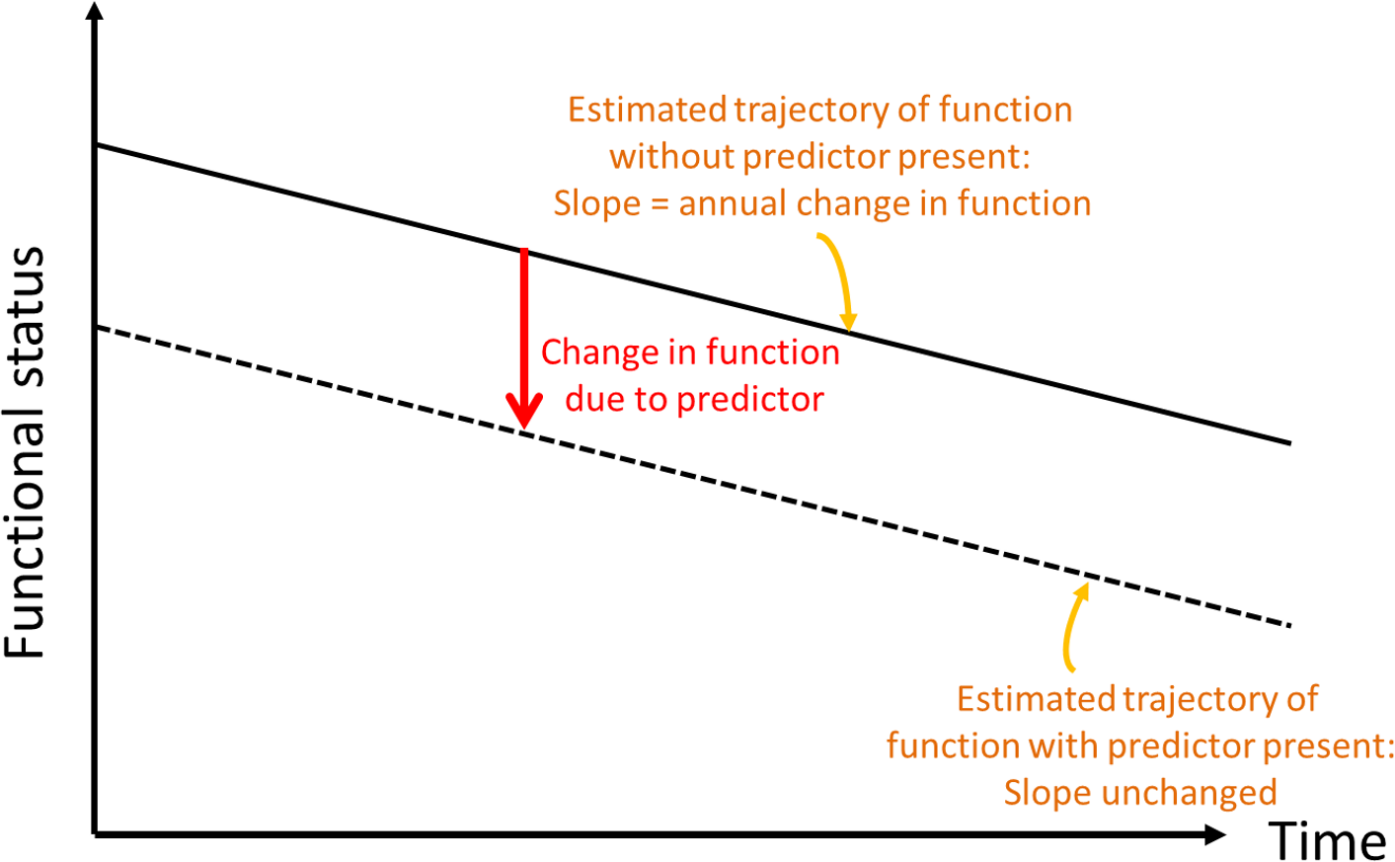
Conceptual depiction of change in baseline functional status.

**Fig 2:**
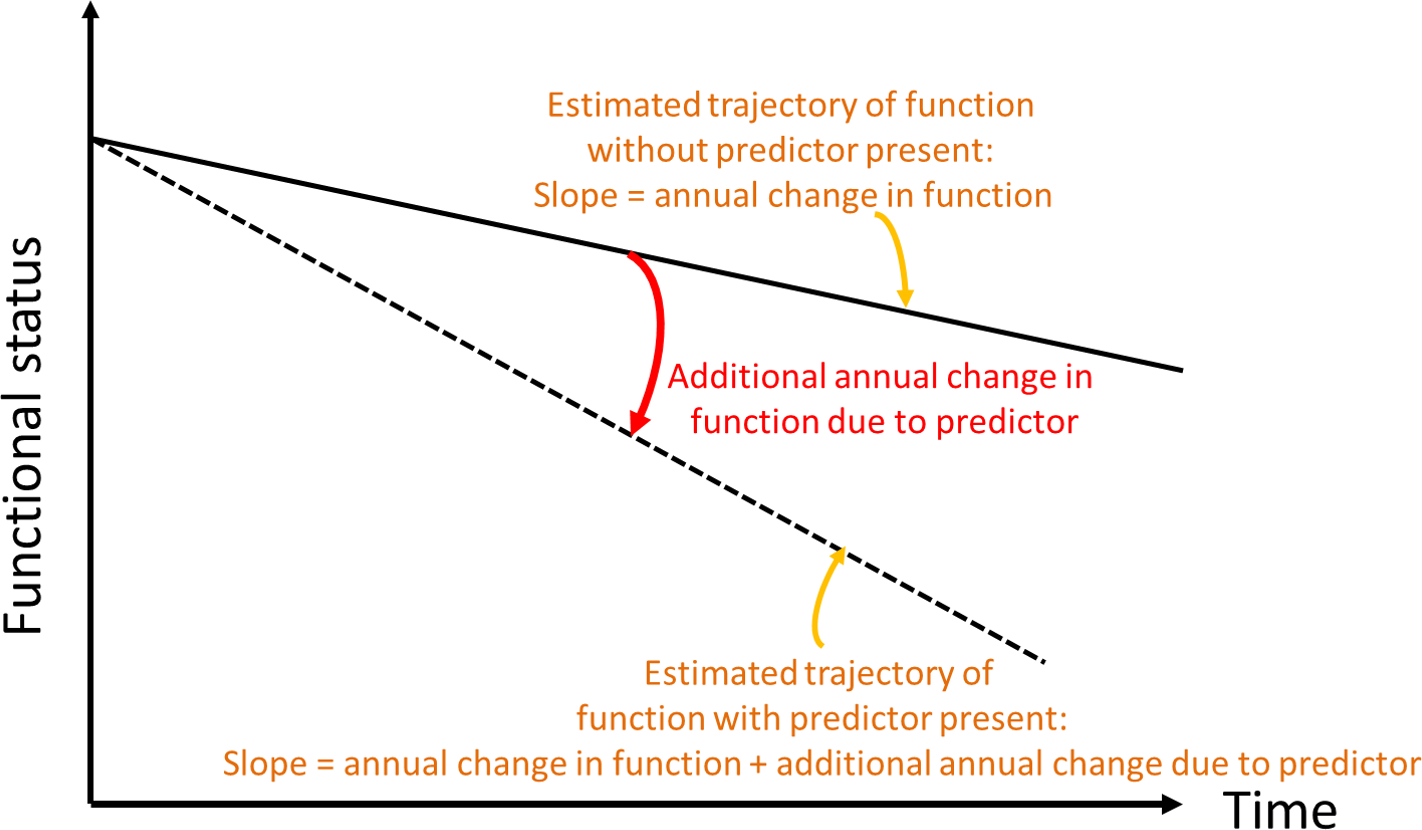
Conceptual depiction of change in slope of functional trajectory.

We then analyzed associations of whole brain WMHV with baseline BI (Fig 1) and slope of decline over time (Fig 2). Due to within-individual correlations among repeated BI measures, regression models using generalized estimating equations (GEEs) with an identity link function were used to assess the association between whole brain WMHV and repeated measurements of BI, adjusting sequentially for: baseline demographic variables, medical risk factors, smoking and alcohol use and physical activity, social variables, and cognitive/mood factors, as defined above.

In order to assess whether MRI variables were associated with change in outcomes over time (slope, Fig 2), we included interaction terms between time of follow-up assessment and the main predictor variable. We used quasi-likelihood under the independence model criterion (QIC) as the model selection criterion after considering candidate final models. Various model diagnostics, including tests of linearity, residual plots, and goodness of fit measures, were used to evaluate the final model. There was no evidence to suggest lack of linearity of BI trajectories in the final models. As a working correlation structure for the GEE models, we chose the exchangeable (intraclass) structure and compared the QIC obtained with this model with one using the unstructured working correlation structure. In order to assess whether interval vascular events were implicated in the trajectory of functional status, we ran a second set of models in which stroke and MI were included as time-varying covariates. We tested whether the relationship between whole brain WMHVs and functional status remained even after adjusting for these events. Using the fully adjusted model, we created a graph of functional trajectories for exemplars with mean age, BMI, MMSE, and Spitzer QOL score; we show 5 estimated trajectories, based on amount of whole brain WMHV: minimum, first quartile, median, third quartile, and maximum.

Next, we examined whether the relationship between whole brain WMHVs and functional status differed for mobility (transfers, mobility, and stair use) and non-mobility (feeding, bathing, grooming, dressing, bowels, bladder, and toilet use) BI domains, which we analyzed separately in unadjusted and fully adjusted models. We also compared trajectories among those with BI 95 or 100 at baseline versus those with baseline BI <95 in order to see if the association was independent of baseline function. The primary models above were additionally adjusted for presence or absence of SBI. Finally, we incorporated mortality into the outcome by performing proportional hazards regression of the relationship between standardized whole brain WMHV and time to the first occurrence of BI score of 60 or death in unadjusted and fully adjusted models. SAS version 9.3 (Cary, NC) was used for all analyses.

## Results

**Fig 3:**
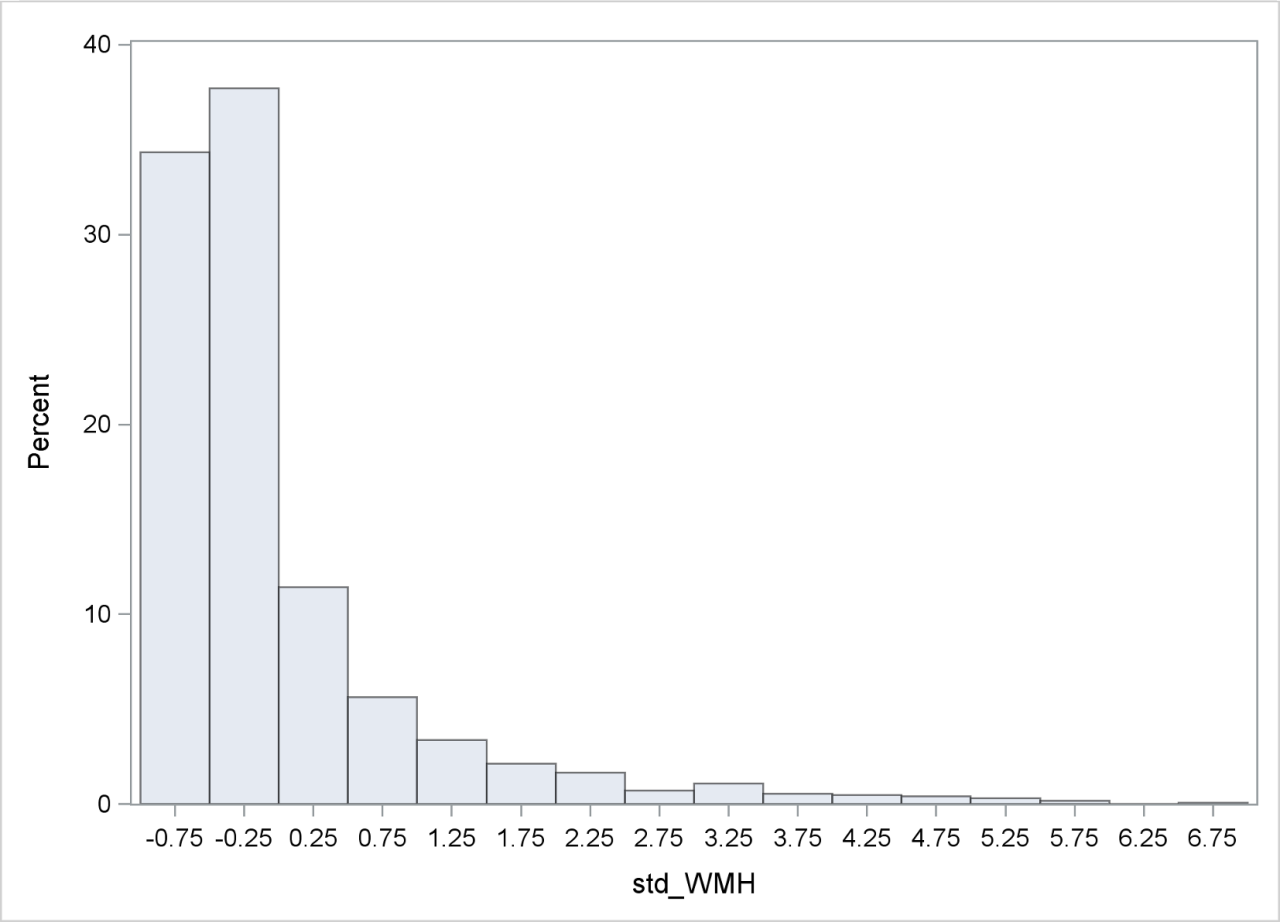
Distribution of WMHVs. **WMHV as percentage of TCV.** BP, blood pressure; CVD, cardiovascular disease; TCV, total cranial volume; WMHV, white matter hyperintensity volume.

Table 1 shows baseline characteristics of the cohort. The mean age was 64.5 years; the cohort was predominantly female (60.5%) and Hispanic (65.7%); and the prevalence of diabetes was 19.0%, hypercholesterolemia 61.8%, and coronary artery disease 13.7%. There were 1,136 individuals (88.8%) who had BI of 95 or 100 at baseline. Mean whole brain WMHVs (as percentage of TCV) was 0.68% (SD 0.84, median 0.36; distribution of whole brain WMHV values in Fig 3), and 193 (15.6%) had SBI. Mean follow-up time per person, from MRI to last follow-up assessment, was 7.3 years (SD 2.1). Out of 1,258 participants with >1 follow-up assessment after MRI, 818 (64.6%) experienced a decline in BI of at least 5 points during follow-up. Of 1,136 participants with BI of 95 or 100 at baseline, 690 (61.7%) experienced a decline in BI. Through the end of 2014, there were 53 first definite and probable MIs during follow-up, and 64 first strokes (59 infarcts, 3 intracerebral hemorrhages, and 2 subarachnoid hemorrhages).

**Table 1:** Baseline characteristics of the cohort.

Characteristic	No. (percent)*
**Number of participants**	1,290 (36.9)
Age, mean (SD), y	64.5 (8.4)
Body mass index, mean (SD), kg/m^2^	28.0 (4.8)
Male	510 (39.5)
Race-ethnicity:	
• Non-Hispanic white	• 191 (14.8)
• Non-Hispanic black	• 223 (17.3)
• Hispanic	• 847 (65.7)
• Other	• 29 (2.3)
Received at least high school education	592 (45.9)
Marital status, married	543 (42.1)
Health insurance	
• Medicaid or no insurance	• 613 (47.5)
• Medicare or private insurance	• 677 (52.5)
Hypertension	861 (66.7)
Alcohol consumption:	
• Never Drank	• 264 (20.5)
• Past Drinker	• 256 (19.8)
• Light Drinker	• 163 (12.6)
• Moderate Drinker	• 530 (41.1)
• Intermediate Drinker	• 49 (3.8)
• Heavy Drinker	• 28 (2.2)
Physical activity:	
• None	• 564 (44.3)
• Any	• 710 (55.7)
Diabetes mellitus	245 (19.0)
Smoking:	
• Never	• 612 (47.4)
• Former	• 496 (38.5)
• Current	• 182 (14.1)
Hypercholesterolemia	797 (61.8)
History of coronary heart disease	177 (13.7)
Hamilton depression scale score, mean (SD), *n* = 1,089	3.1 (3.8)
Mini mental state score, mean (SD), *n* = 1,289	26.7 (3.3)
Spitzer QOL index score, *n* = 1,090	9.3 (1.0)
Number of people known well enough to visit with in their homes:	
• None	• 36 (2.8)
• 1 or 2	• 124 (9.6)
• 3 or 4	• 263 (20.4)
• 5 or more	• 867 (67.2)
Number of years living in community	25.3 (14.9)

**Abbreviations:** SD, standard deviation; QOL, quality of life.

*unless otherwise indicated

**Fig 4:**
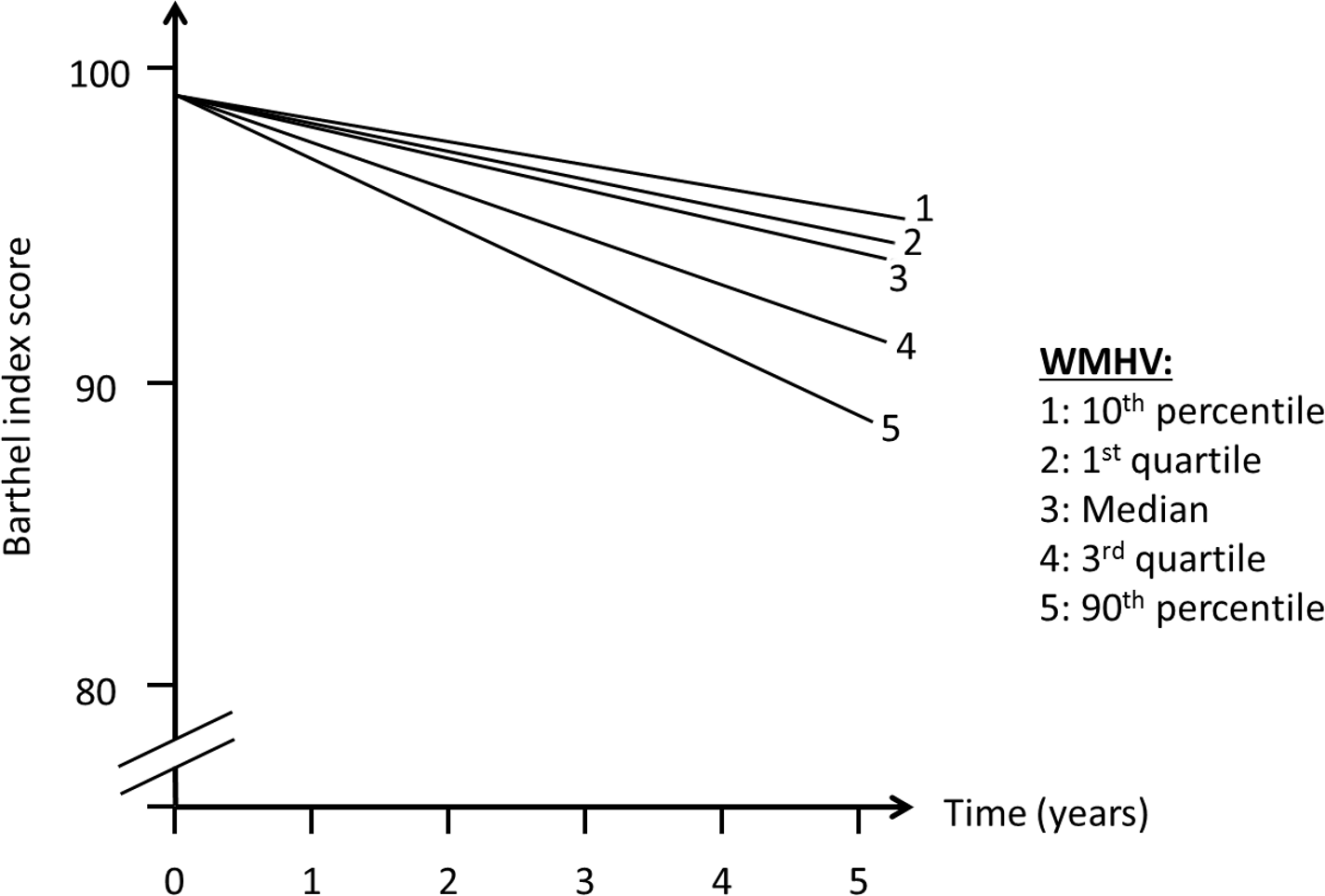
Estimated trajectories of functional status, by amount of white matter hyperintensity. WMHV, white matter hyperintensity volume.

There was an annual decline in functional status overall of −1.12 BI points per year (95% CI −1.28, −0.97) in an unadjusted model and −1.04 points per year (95% CI −1.20, −0.88) in a fully adjusted model (Table 2). Standardized whole brain WMHV was associated with accelerated functional decline, with −0.82 additional BI points per year (95% CI −1.06, −0.57) per SD greater whole brain WMHV in an unadjusted model and −0.74 additional points per year (95% CI −0.99, −0.49) in a fully adjusted model. The magnitude of this effect varied little with additional adjustment, even after adjusting for intervening clinical stroke and MI. Whole brain WMHV had a consistent effect on mobility and non-mobility domains of the BI (Table 3), proportional to the portion of the BI comprising each domain. In adjusted models, whole brain WMHV was not associated with baseline BI. Fig 4 shows estimated trajectories of functional status based on fully adjusted models (Table 2), stratified by whole brain WMHV.

**Table 2:** Unadjusted and adjusted models of the association between standardized whole brain white matter hyperintensity volume (WMHV/TCV*100) and functional status.

Variable	Change in BI score	95% CI	*p*-value
**Unadjusted model:**			
Change in BI with 1 SD increase in WMH	−1.08	−2.08, −0.09	0.03
Annual change in BI at mean baseline WMHV	−1.12	−1.28, −0.97	< .0001
Additional annual change with 1 SD increase in WMH	−0.82	−1.06, −0.57	< .0001
**Adjusted for demographics†:**			
Change in BI with 1 SD increase in WMH	0.13	−0.92, 1.18	0.8
Annual change in BI at mean baseline WMHV	−1.14	−1.30, −0.98	< .0001
Additional annual change with 1 SD increase in WMH	−0.82	−1.07, −0.57	< .0001
Adjusted for vascular risk factors*:			
Change in BI with 1 SD increase in WMH	0.30	−0.89, 1.48	0.6
Annual change in BI at mean baseline WMHV	−1.17	−1.34, −1.00	< .0001
Additional annual change with 1 SD increase in WMHs	−0.78	−1.04, −0.52	< .0001
Adjusted for social variables**:			
Change in BI with 1 SD increase in WMHs	0.35	−0.83, 1.52	0.6
Annual change in BI at mean baseline WMHV	−1.18	−1.34, −1.01	< .0001
Additional annual change with 1 SD increase in WMHs	−0.78	−1.04, −0.52	< .0001
**Adjusted for stroke and MI‡:**			
Change in BI with 1 SD increase in WMHs	0.59	−0.50, 1.68	0.3
Annual change in BI at mean baseline WMHV	−1.04	−1.20, −0.88	< .0001
Additional annual change with 1 SD increase in WMHs	−0.74	−0.99, −0.49	< .0001

**Abbreviations:** BI, Barthel index; CI, confidence interval; MI, myocardial infarction; TCV, total cranial volume; WMH, white matter hyperintensity; WMHV, white matter hyperintensity volume.

† adjusted for age at time of MRI, sex, race-ethnicity

* additionally adjusted for: diabetes, hypertension, coronary artery disease, hypercholesterolemia, physical activity, alcohol use, smoking, and body mass index at the time of MRI

** additionally adjusted for: marital status, insurance, number of friends, and years lived in the community, mini-mental state score, Spitzer QOL index, depression at baseline

‡ additionally adjusted for stroke and MI occurring during follow-up, as time-varying covariates

**Table 3:** Unadjusted and adjusted models of the association between standardized whole brain white matter hyperintensity volume (WMHV/TCV) and functional status, stratified by mobility versus non-mobility domains.

	Mobility domain			Non-mobility domain		
Variable	Change in BI score	95% CI	*p*-value	Change in BI score	95% CI	*p*-value
**Unadjusted model:**						
Change in BI with 1 SD increase in WMHs	−0.55	−0.98, −0.12	0.013	−0.55	−1.16, 0.06	0.08
Annual change in BI at mean baseline WMHV	−0.49	−0.55, −0.42	< .0001	−0.63	−0.73, −0.54	< .0001
Additional annual change with 1 SD increase in WMHs	−0.31	−0.41, −0.21	< .0001	−0.49	−0.65, −0.34	< .0001
**Adjusted for demographics†:**						
Change in BI with 1 SD increase in WMH	0.05	−0.39, 0.49	0.8	0.07	−0.57, 0.71	0.8
Annual change in BI at mean baseline WMHV	−0.49	−0.56, −0.43	< .0001	−0.64	−0.74, −0.54	< .0001
Additional annual change with 1 SD increase in WMHs	−0.32	−0.42, −0.22	< .0001	−0.50	−0.65, −0.34	< .0001
Adjusted for vascular risk factors*:						
Change in BI with 1 SD increase in WMHs	0.16	−0.34, 0.66	0.5	0.13	−0.60, 0.86	0.7
Annual change in BI at mean baseline WMHV	−0.51	−0.58, −0.44	< .0001	−0.66	−0.76, −0.55	< .0001
Additional annual change with 1 SD increase in WMHs	−0.30	−0.40, −0.19	< .0001	−0.47	−0.64, −0.31	< .0001
Adjusted for social variables**:						
Change in BI with 1 SD increase in WMHs	0.19	−0.30, 0.68	0.5	0.15	−0.57, 0.88	0.7
Annual change in BI at mean baseline WMHV	−0.51	−0.58, −0.44	< .0001	−0.66	−0.77, −0.56	< .0001
Additional annual change with 1 SD increase in WMHs	−0.30	−0.40, −0.19	< .0001	−0.47	−0.64, −0.31	< .0001
**Adjusted for stroke and MI‡:**						
Change in BI with 1 SD increase in WMHs	0.29	−0.18, 0.76	0.2	0.29	−0.38, 0.96	0.4
Annual change in BI at mean baseline WMHV	−0.45	−0.52, −0.38	< .0001	−0.58	−0.69, −0.48	< .0001
Additional annual change with 1 SD increase in WMHs	−0.28	−0.38, −0.18	< .0001	−0.45	−0.61, −0.30	< .0001

**Abbreviations:** BI, Barthel index; CI, confidence interval; MI, myocardial infarction; TCV, total cranial volume; WMH, white matter hyperintensity; WMHV, white matter hyperintensity volume.

NOTE: The mobility domain includes transfers, mobility, and stair use; the non-mobility domain includes feeding, bathing, grooming, dressing, bowels, bladder, and toilet use

† adjusted for age at time of MRI, sex, race-ethnicity

* additionally adjusted for: diabetes, hypertension, coronary artery disease, hypercholesterolemia, physical activity, alcohol use, smoking, and body mass index at the time of MRI

** additionally adjusted for: marital status, insurance, number of friends, years lived in the community, mini-mental state score, Spitzer QOL index and depression

‡ additionally adjusted for stroke and MI occurring during follow-up, as time-varying covariates

We compared functional trajectories among those with a baseline BI score of 95 or 100 (*n* = 1,136) versus those with baseline BI < 95 (*n* = 144, Table 4). Although an interaction term between baseline BI score and annual change in BI was significant (*p* <0.0001), in both groups, whole brain WMHV was associated with an additional annual decline of −0.68 points per year (95% CI −0.94, −0.41) among those with baseline BI of 95 or 100 and of −0.86 points per year (95% CI −1.49, −0.23) among those with baseline BI <95.

**Table 4:** Associations between whole brain WMHV and functional status stratified by baseline BI score, in adjusted models.

	Among those with BI < 95 at baseline (*n* = 144)			Among those with BI ≥ 95 at baseline (*n* = 1,136)		
	Change in BI score	95% CI	*p*-value	Change in BI score	95% CI	*p*-value
Change in BI with 1 SD increase in WMHs	−1.76	−4.91, 1.38	0.3	1.17	0.14, 2.20	0.026
Annual change in BI at mean baseline WMHV	−2.10	−2.83, 1.37	<0.0001	−0.94	−1.10, −0.78	< .0001
Additional annual change with 1 SD increase in WMH	−0.86	−1.49, −0.23	0.008	−0.68	−0.94, −0.41	< .0001

**Abbreviations:** BI, Barthel index; CI, confidence interval; SD, standard deviation; WMH, white matter hyperintensity; WMHV, white matter hyperintensity volume.

Models are adjusted for: age at the time of MRI, sex, race-ethnicity, diabetes, hypertension, hypercholesterolemia, physical activity, alcohol use, body mass index, insurance status, stroke and myocardial infarction occurring during follow-up, and MMSE score.

Next, the primary models were additionally adjusted for SBI, in order to assess whether the association between whole brain WMHV and functional trajectories was independent of the presence of SBI. In the fully adjusted model, there was an annual decline in functional status overall of −1.04 BI points per year (95% CI −1.20, −0.88), and standardized whole brain WMHV was still associated with accelerated functional decline, with −0.74 additional BI points per year (95% CI −0.99, −0.49) per SD higher whole brain WMHV.

When we incorporated mortality into the outcome using proportional hazards regression, there was an increased hazard of the occurrence of a BI of 60 or death with higher whole brain WMHV, with HR of 1.52 per SD increase in whole brain WMHV (95% CI 1.42,1.64) in an unadjusted model and 1.31 (95% CI 1.19,1.44) in a fully adjusted model.

## Discussion

In this large population-based MRI study with over 7 years of follow-up and annual functional measurements, there was a strong, consistent, and independent association of greater whole brain WMHV with accelerated decline in function over time equivalent to an almost doubling of the mean annual decline per SD whole brain WMHV increase. This decline was also seen when mobility and non-mobility domains of the BI were examined as separate outcomes and even after adjusting for SBI. Also, these associations were seen even among those with no disability at baseline, which emphasizes the “subclinical” nature of these predictors and yet their strong predictive power on trajectories of functional status. In a sensitivity analysis that incorporated mortality, we found that every SD increase in whole brain WMHV was associated with approximately 30% increased hazard of poor functional outcome or death. Although there was an unadjusted association between greater whole brain WMHV and lower baseline BI score, we did not observe an association between whole brain WMHV and baseline BI in adjusted models. This suggests that demographic variables, primarily age, confounded the relationship between whole brain WMHV and baseline functional status.

Several studies have examined associations between patient-centered outcomes and WMHV. In a prior analysis in the NOMAS MRI cohort [21], greater whole brain WMHV and smaller TCV were associated with poorer performance in learning a list of words and greater decline in global cognition [22]. The current analysis expands upon this previous research by analyzing longitudinal trends of repeated measures of functional status and confirming a long-term effect of whole brain WMHV on functional decline. Among 619 participants [23], risk of transition to ≥2 ADL impairments at 1 year was higher with more WMHV. After a mean of 2.42 years of follow-up, these trends were maintained [24], with an HR for transition to disability or death of 2.36 (95% CI 1.65–3.81). When gait and balance were examined yearly over 3 years of follow-up, more WMHV predicted greater decline over time, especially among older adults [25]. Among 287 community-dwelling individuals aged 70–90 years [26], greater WMHV was associated with physical decline over 1 year (odds ratio [OR] 3.02, 95% CI 1.02–8.95). Among 99 individuals 75–89 years of age, global WMHV was associated with urinary incontinence, mobility deficits, and executive dysfunction [27].

Multiple prior studies have shown relationships between subclinical ischemic injury and patient-centered outcomes. However, a minority of prior studies has examined trajectories of these outcomes over time, describing not only change between 2 time points but also slopes of change over time with multiple repeated measures per individual. Trajectory analysis is more sensitive to the course of change in outcomes over time and can reliably estimate this slope, thereby describing the natural history of functional status and factors that influence it. Also, we controlled for adjudicated vascular outcomes occurring during follow-up, allowing the estimation of associations independent of clinical events.

WMHV, SBI, microbleeds, and atrophy have been associated with declines in gait speed, cadence of gait, and stride length [28,29], which would affect ADLs dependent on mobility aspects of functioning. WMHV and its progression have been associated with neurological examination findings such as gait and stance abnormalities, upper motor signs, and slowing of finger taps [30], and the presence and number of neurological deficits have an independent impact on performance of ADLs [31]. Silent deep infarcts and WMHV have been associated with gait variability, which has been associated with falls and disability [32].

Strengths of this study include the large population-based cohort, accurate assessment of follow-up events, minimal loss to follow-up, state-of-the-art imaging and measurement of subclinical brain vascular disease, and repeated measures of functional outcomes that allow trajectory analysis. A limitation is that NOMAS participants enrolled in the MRI cohort were able to return for follow-up and undergo MRI, reflecting a healthy survivor bias, which may have reduced power to detect declines in functional status and resulted in the lack of association between whole brain WMHV and baseline functional status. Also, in terms of intervening conditions or events that could affect functional trajectories, we focused on the vascular events of stroke and MI but did not adjust for nonvascular intervening conditions. Similarly, we did not measure or assess time-dependent alterations on MRI in measures such as atrophy, which could have an effect on functional status over time.

There are several implications of this research. With an exclusive focus on short-term events—traditionally the approach of many observational studies and clinical trials—the long-term declines seen in these analyses would be missed, and the burden of disease would be underestimated. Also, few studies have analyzed not only single time points but trajectories over time, which allows for a more detailed characterization of the relationship between whole brain WMHV and disability trajectories. This research highlights the likely central role that “subclinical” disease plays in functional ability and health.

## Supporting information

S1 Text STROBE checklist. (DOC)

S2 Text Original analysis plan from Dr. Dhamoon’s dissertation proposal. (DOC)

## Abbreviations

ADL: activity of daily living
BI: Barthel index
CSF: cerebral spinal fluid
CUMC: Columbia University Medical Center
CVD: cardiovascular disease
GEE: generalized estimating equation
MI: myocardial infarction
MMSE: mini-mental state examination
NOMAS: Northern Manhattan Study
OR: odds ratio
QIC: quasi-likelihood under the independence model criterion
QOL: quality of life
SBI: subclinical brain infarct
SD: standard deviation
STROBE: Strengthening the Reporting of Observational Studies in Epidemiology
TCV: total cranial volume
WMH: white matter hyperintensity
WMHV: white matter hyperintensity volume
