# Supplementary material for: Cerebral white matter disease and functional decline in older adults from the Northern Manhattan Study: A longitudinal cohort study
Source: PLoS Med. 2018 Mar 20;15(3):e1002529. doi: 10.1371/journal.pmed.1002529 (PMC5860694; doi:10.1371/journal.pmed.1002529)
Supplement: S1 Text — (DOC) [file pmed.1002529.s001.doc]

| **Item** | **Item number** | **STROBE Guidelines** | **LOCATION:** |  |
| --- | --- | --- | --- | --- |
| **Title and abstract** | 1 | (a) Indicate the study’s design with a commonly used term in the title or the abstract | Title is: Association between cerebral white matter disease and functional decline in older adults: a cohort study |  |
|  |  | (b) Provide in the abstract an informative and balanced summary of what was done and what was found | Abstract |  |
| **Introduction** |  |  |  |  |
| Background rationale | 2 | Explain the scientific background and rationale for the investigation being reported | First 2 paragraphs |  |
| Objectives | 3 | State specific objectives, including any pre-specified hypotheses | Second paragraph of Background |  |
| **Methods** |  |  |  |  |
| Study design | 4 | Present key elements of study design early in the paper | First paragraph of Methods |  |
| Setting | 5 | Describe the setting, locations, and relevant dates, including periods of recruitment, exposure, follow-up, and data collection | First 5 paragraphs of Methods |  |
| Participants | 6 | (a) Cohort study—Give the eligibility criteria, and the sources and methods of selection of participants. Describe methods of  follow-up  Case-control study—Give the eligibility criteria, and the sources and methods of case ascertainment and control selection. Give  the rationale for the choice of cases and controls  Cross-sectional study—Give the eligibility criteria, and the sources and methods of selection of participants | Methods |  |
|  |  | (b) Cohort study—For matched studies, give matching criteria and number of exposed and unexposed  Case-control study—For matched studies, give matching criteria and the number of controls per case | N/A |  |
| Variables | 7 | Clearly define all outcomes, exposures, predictors, potential confounders, and effect modifiers. Give diagnostic criteria, if applicable | Methods |  |
| Data source/measurement | 8 | For each variable of interest, give sources of data and details of methods of assessment (measurement).  Describe comparability of assessment methods if there is more than one group | Methods |  |
| Bias | 9 | Describe any efforts to address potential sources of bias | Methods, last paragraph |  |
| Study size | 10 | Explain how the study size was arrived at | Methods, first paragraph |  |
| Quantitative variables | 11 | Explain how quantitative variables were handled in the analyses. If applicable, describe which groupings were chosen, and why | Methods |  |
| Statistical methods | 12 | (a) Describe all statistical methods, including those used to control for confounding | Methods, Statistical Analysis |  |
|  |  | (b) Describe any methods used to examine subgroups and interactions | Methods, Statistical Analysis |  |
|  |  | (c) Explain how missing data were addressed | Methods, Statistical Analysis |  |
|  |  | (d) Cohort study—If applicable, explain how loss to follow-up was addressed  Case-control study—If applicable, explain how matching of cases and controls was addressed  Cross-sectional study—If applicable, describe analytical methods taking account of sampling strategy | Methods, Follow-up |  |
|  |  | (e) Describe any sensitivity analyses | Methods, last paragraph |  |
| *Validity/reliability of measurement and internal/external validation* |  |  |  |  |
| **Results** |  |  |  |  |
| Participants | 13 | (a) Report the numbers of individuals at each stage of the study—e.g., numbers potentially eligible, examined for eligibility, con-  firmed eligible, included in the study, completing follow-up, and analysed | Methods |  |
|  |  | (b) Give reasons for non-participation at each stage |  |  |
|  |  | (c) Consider use of a flow diagram |  |  |
| Descriptive data | 14 | (a) Give characteristics of study participants (e.g., demographic, clinical, social) and information on exposures and potential con-  founders | Table 1 |  |
|  |  | (b) Indicate the number of participants with missing data for each variable of interest | Table 1 |  |
|  |  | (c) Cohort study—Summarise follow-up time (e.g., average and total amount) | Results |  |
| *Distribution of biomarker measurement* |  |  |  |  |
| Outcome data | 15 | Cohort study—Report numbers of outcome events or summary measures over time  Case-control study—Report numbers in each exposure category, or summary measures of exposure  Cross-sectional study—Report numbers of outcome events or summary measures | Results |  |
| Main results | 16 | (a) Give unadjusted estimates and, if applicable, confounder-adjusted estimates and their precision (e.g., 95% confidence interval).  Make clear which confounders were adjusted for and why they were included | Tables 2-4 |  |
|  |  | (b) Report category boundaries when continuous variables were categorized | Methods |  |
|  |  | (c) If relevant, consider translating estimates of relative risk into absolute risk for a meaningful time period | N/A |  |
| Other analyses | 17 | Report other analyses done—e.g., analyses of subgroups and interactions, and sensitivity analyses | Results |  |
| **Discussion** |  |  |  |  |
| Key results | 18 | Summarise key results with reference to study objectives | Discussion, first paragraph |  |
| Limitations | 19 | Discuss limitations of the study, taking into account sources of potential bias or imprecision. Discuss both direction and magnitude  of any potential bias | Discussion, second to last paragraph |  |
| Interpretation | 20 | Give a cautious overall interpretation of results considering objectives, limitations, multiplicity of analyses, results from similar studies, and other relevant evidence | Discussion |  |
| Generalisability | 21 | Discuss the generalisability (external validity) of the study results | Discussion |  |
| **Other information** |  |  |  |  |
| Funding | 22 | Give the source of funding and the role of the funders for the present study and, if applicable, for the original study on which the  present article is based | Funding statement |  |
| Ethics |  |  |  |  |
