# Supplementary material for: Cerebral white matter disease and functional decline in older adults from the Northern Manhattan Study: A longitudinal cohort study
Source: PLoS Med. 2018 Mar 20;15(3):e1002529. doi: 10.1371/journal.pmed.1002529 (PMC5860694; doi:10.1371/journal.pmed.1002529)
Supplement: S2 Text — (DOC) [file pmed.1002529.s002.doc]

**Specific Aims**

Stroke is the leading cause of disability^1^ and a significant cause of cognitive impairment and depression in the immediate post-stroke period.^2-4^ Stroke is traditionally seen as a discrete event, and it is assumed that, following the 3-6 month recovery period after stroke, functional status would more or less stabilize unless recurrent events occur. Indeed, the short-term effects of stroke on disability are well-described, but the long-term course of functional status before and after stroke is less clear.^5, 6^ In contrast to this traditional view of stroke, there is growing evidence that a paradigm may be more appropriate in which the effect of cerebrovascular disease on disability is viewed in a continuous, ongoing manner. In other words, stroke may be more effectively considered as an ongoing, chronic condition with effects on function, instead of a discrete event. For example, stroke is caused by conditions that may have an ongoing and cumulative effect on vessel dysfunction, including vascular risk factors and inflammatory states. In addition to causing recurrent strokes, such processes cause subclinical infarcts and white matter disease that may reduce functional status over the long term.^7, 8^ It is also possible that individual strokes cause injury to the brain that lead to a chronic and degenerative process with progressive damage, dysfunction, and functional decline.

Hypothesis #1: Serum inflammatory biomarkers and cerebral white matter disease independently predict worse functional status in NOMAS in those free of stroke at baseline.

Specific Aim #1: a) To determine whether levels of serum inflammatory biomarkers measured at the time of enrollment (interleukin-6, tumor necrosis factor alpha receptor, C-reactive protein, lipoprotein-associated phospholipase) are associated with lower Barthel index (BI) scores and a steeper slope of decline in a multiethnic cohort, using multivariable regression and adjusting for baseline demographic characteristics (age, sex), vascular risk factors (diabetes, hypertension, hypercholesterolemia), behavioral factors (smoking and alcohol use), social variables (marital status, insurance status, number of friends), and cognitive factors (depressed mood, performance on mini-mental state examination).

b) In a subset of the cohort in which study brain MRI was performed, to determine whether volumes of cerebral white matter disease and subclinical brain infarcts are associated with BI, using the same approach as (a).

**1.C.6. Study outcome**

The BI, developed in 1965^9^ and later modified,^10^ measures an individual’s performance in 10 ADLs and has been extensively used in stroke observational studies and clinical trials as a measure of post-stroke disability.^11^ The scale ranges from 0 to 100 in 5-point increments, with 100 indicating normal physical functioning. Previous research has demonstrated the reliability of phone assessments of function using the BI.^12^ Although it is an ordinal scale, recent research has advocated analyzing the scale as a continuous variable due to increased power to detect associations, ability to describe the course of change over time in linear form, and avoidance of potential misclassification due to crude categorization.^13-15^

**1.C.7. Explanatory variables**

1. Inflammatory biomarkers: The distributions of all 4 biomarkers (IL-6, TNF-R, CRP, and LpPLA(2)) will be determined. The functional relationship between the different biomarkers and BI scores will be explored using restricted cubic splines. Depending on the distributions, simple log transformations may be employed to satisfy the linearity requirements of the model. Otherwise, quartiles of each will be used as categorical variables using the lowest quartile as the referent group. Additionally, CRP will be categorized according to the three CDC/AHA risk stratification levels: <1 mg/L; 1-3 mg/L; and >3 mg/L.^16^

2. MRI white matter disease: Total WMH volumes will be calculated after correcting for total cranial volume and will be treated as a continuous (log transformed) and categorical variable (quartiles) consistent with previous analyses in NOMAS.^17-19^ We will incorporate other proposed methods for categorizing WMH as a dichotomous variable based on age-normalized total WMH volume.^20, 21^ SBIs will be defined as lesions greater than 3 mm in size, distinct from the circle of Willis in the basal ganglia, and of similar intensity as cerebrospinal fluid. The location and size of SBIs will also be recorded.^22^ Dr. Clinton Wright, Chair of the Neuroimaging Committee of NOMAS, will provide assistance with variable definition and analysis on biweekly conference calls.

**1.C.8. Covariates**

The proposed etiologic model is presented in Figure 4 (note that this is not intended to be a directed acyclic graph). Vascular risk factors have a direct impact on vessel dysfunction (including subclinical infarcts and clinical strokes) as well as an indirect effect, mediated through systemic inflammation. Systemic inflammatory states cause elevations in serum biomarkers, which are measured in order to quantify the degree of inflammation present. Other factors, indicated by ‘U_1_’ and not measured in this analysis, also influence systemic inflammation. A different set of unmeasured factors, U_2_, influence vascular dysfunction and stroke. Vessel dysfunction is detected, in part, by structural brain changes measured on brain MRI as subclinical brain infarcts and white matter disease. Vascular dysfunction causes impairment in blood flow and structural damage to the brain, which causes cognitive and physical impairments. These impairments cause impaired functional status by affecting an individual’s performance in ADLs and IADLs.


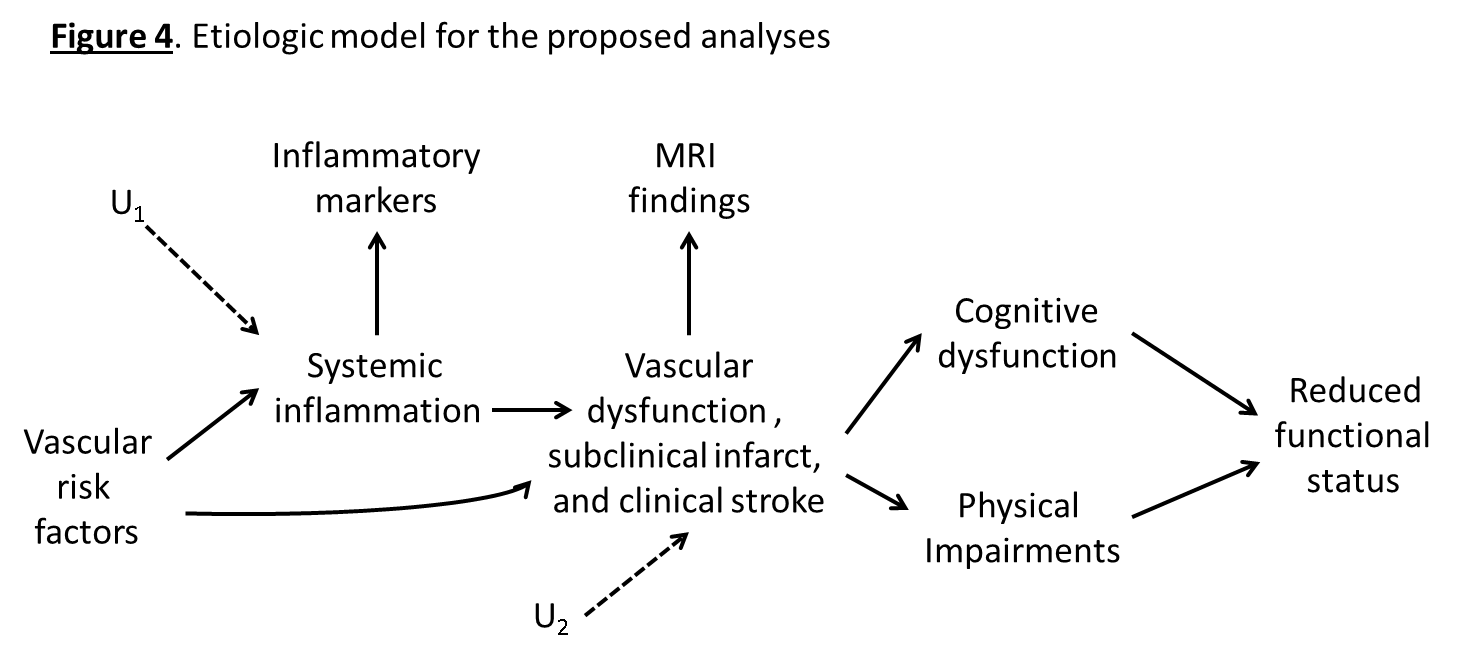
 All analytic models will be adjusted for the following variables: age, sex, body mass index (body weight in kilograms divided by the square of height in meters), self-reported hypercholesterolemia, diabetes mellitus (defined by self-report, fasting blood glucose level >126 mg/dL, or insulin/oral hypoglycemic use), hypertension (defined as a systolic blood pressure recording >140 mmHg or a diastolic blood pressure recording >90 mm Hg based on the average of two blood pressure measurements or the patient's self-report of a history of hypertension or antihypertensive use), smoking (defined as either nonsmoker or smoker within the last year), alcohol use (with moderate alcohol use classified as 1 drink/month to 2 drinks/day), social variables (marital status, insurance status [classified as uninsured, Medicaid, Medicare, or private insurance], median income of residential zip code, number of friends [individuals whom the participant knows well enough to visit in their homes], years living in the community), and cognitive factors (depressed mood, and performance on mini-mental state examination [analyzed as a continuous variable]).

**1.C.9. Statistical analysis**

1. Inflammatory biomarkers: The goal of this analysis is to determine whether levels of serum inflammatory biomarkers (IL-6, TNF-R, CRP, LpPLA(2)) are associated with BI and a steeper slope of decline over time. We will first calculate the distribution of main explanatory variables, baseline covariates, and BI. Then, each biomarker exposure will be analyzed separately. Due to correlations among repeated measures of outcomes in the same individual, regression models based upon generalized estimating equations (GEE) with an identity link function will be used to assess the association between main explanatory variables and repeated measurements of BI, adjusting for baseline demographic variables (age, sex), medical risk factors (BMI, diabetes, hypertension, hypercholesterolemia), smoking and alcohol use, social variables (marital status, insurance status, number of friends, number of years in the community, median income of residential zip code), and cognitive factors (depressed mood, performance on mini-mental state examination).

In model building, we will sequentially add groups of variables in a pre-specified manner based upon the standards of the field. Specifically, the first model will include no covariates, and successive models will include demographic variables, vascular risk factors, social variables, and cognitive and mood factors. To assess whether the main explanatory variables are associated with the slope of change in outcomes over time, we will include interaction terms between time of follow-up assessment and the variable. All significant interactions with time will be included in the final model. We will use QIC for GEE models and AIC for mixed models as the model selection criteria after considering candidate final models. Various model diagnostics including tests of linearity, residual plots, and goodness of fit measures will be used to evaluate the final model. As a working correlation structure for the GEE models we will choose the exchangeable (intraclass) structure and compare the QIC obtained with this model with one using the unstructured working correlation structure. We will choose the model with the lower QIC as the final model.

To assess whether interval vascular events such as clinical stroke and MI are implicated in the trajectory of functional status, we will run a second set of models in which time of follow-up will be censored at the time of stroke and MI. The parameter estimates of the primary terms as well as interaction terms with time will be qualitatively compared to the model without censoring to assess for changes in parameter estimates. In a separate analysis, we will assess whether interval non-stroke and non-MI hospitalization is related to changes in functional status, using a similar strategy as above.

We will also pursue an alternative modeling strategy using mixed models. For the mixed models approach, we will first calculate intraclass correlation coefficients using an ‘empty’ or ‘random intercept only’ model to determine the proportion of variance that is due to between-person variation. We will then determine whether there is an effect of time on average on the outcome of functional status by determining whether there is a fixed effect of time. We will also determine whether the average effect of time varies across individuals by determining the random effect of time. We will add covariates in a similar manner as for the analyses described above.

2. MRI white matter disease: The goal of this analysis is to determine whether increasing volumes of cerebral white matter disease and SBI are associated with BI and a steeper slope of decline. Each outcome will be analyzed in separate models, using an approach similar to that outlined under (1) above.

For all analyses, sensitivity analyses for loss to follow-up will be performed in several ways. First, multiple imputation will be used for missing data due to truncation by death. Second, fully conditional pattern-mixture models, which stratify by the time of dropout, will be used to determine whether there is change in the trajectories of functional change found in the primary analyses.^23^ We will not use these models for the primary analysis since they incorporate information about time of death, which cannot be used in a predictive model since an individual’s time of death is not known at baseline.

**References:**

1. Mohr JP, Thompson JL, Lazar RM, Levin B, Sacco RL, Furie KL, et al. A comparison of warfarin and aspirin for the prevention of recurrent ischemic stroke. *N Engl J Med*. 2001;345:1444-1451

2. Duncan PW, Samsa GP, Weinberger M, Goldstein LB, Bonito A, Witter DM, et al. Health status of individuals with mild stroke. *Stroke*. 1997;28:740-745

3. Lai SM, Studenski S, Duncan PW, Perera S. Persisting consequences of stroke measured by the stroke impact scale. *Stroke*. 2002;33:1840-1844

4. Suenkeler IH, Nowak M, Misselwitz B, Kugler C, Schreiber W, Oertel WH, et al. Timecourse of health-related quality of life as determined 3, 6 and 12 months after stroke. Relationship to neurological deficit, disability and depression. *J Neurol*. 2002;249:1160-1167

5. Dhamoon MS, Moon YP, Paik MC, Boden-Albala B, Rundek T, Sacco RL, et al. Long-term functional recovery after first ischemic stroke: The northern manhattan study. *Stroke*. 2009;40:2805-2811

6. Dhamoon MS, Moon YP, Paik MC, Boden-Albala B, Rundek T, Sacco RL, et al. Quality of life declines after first ischemic stroke. The northern manhattan study. *Neurology*. 2010;75:328-334

7. Vermeer SE, Koudstaal PJ, Oudkerk M, Hofman A, Breteler MM. Prevalence and risk factors of silent brain infarcts in the population-based rotterdam scan study. *Stroke*. 2002;33:21-25

8. Rosano C, Kuller LH, Chung H, Arnold AM, Longstreth WT, Jr., Newman AB. Subclinical brain magnetic resonance imaging abnormalities predict physical functional decline in high-functioning older adults. *J Am Geriatr Soc*. 2005;53:649-654

9. Mahoney FI, Barthel DW. Functional evaluation: The barthel index. *Md State Med J*. 1965;14:61-65

10. Granger CV, Dewis LS, Peters NC, Sherwood CC, Barrett JE. Stroke rehabilitation: Analysis of repeated barthel index measures. *Arch Phys Med Rehabil*. 1979;60:14-17

11. Sulter G, Steen C, De Keyser J. Use of the barthel index and modified rankin scale in acute stroke trials. *Stroke*. 1999;30:1538-1541

12. Shinar D, Gross CR, Bronstein KS, Licata-Gehr EE, Eden DT, Cabrera AR, et al. Reliability of the activities of daily living scale and its use in telephone interview. *Arch Phys Med Rehabil*. 1987;68:723-728

13. Bath PM, Gray LJ, Collier T, Pocock S, Carpenter J. Can we improve the statistical analysis of stroke trials? Statistical reanalysis of functional outcomes in stroke trials. *Stroke*. 2007;38:1911-1915

14. Saver JL. Optimal end points for acute stroke therapy trials: Best ways to measure treatment effects of drugs and devices. *Stroke*. 2011;42:2356-2362

15. Song F, Jerosch-Herold C, Holland R, Drachler Mde L, Mares K, Harvey I. Statistical methods for analysing barthel scores in trials of poststroke interventions: A review and computer simulations. *Clin Rehabil*. 2006;20:347-356

16. Pearson TA, Mensah GA, Alexander RW, Anderson JL, Cannon RO, 3rd, Criqui M, et al. Markers of inflammation and cardiovascular disease: Application to clinical and public health practice: A statement for healthcare professionals from the centers for disease control and prevention and the american heart association. *Circulation*. 2003;107:499-511

17. Wright CB, Paik MC, Brown TR, Stabler SP, Allen RH, Sacco RL, et al. Total homocysteine is associated with white matter hyperintensity volume: The northern manhattan study. *Stroke*. 2005;36:1207-1211

18. Wright CB, Moon Y, Paik MC, Brown TR, Rabbani L, Yoshita M, et al. Inflammatory biomarkers of vascular risk as correlates of leukoariosis. *Stroke*. 2009;40:3466-3471

19. Wright CB, Festa JR, Paik MC, Schmiedigen A, Brown TR, Yoshita M, et al. White matter hyperintensities and subclinical infarction: Associations with psychomotor speed and cognitive flexibility. *Stroke*. 2008;39:800-805

20. Debette S, Beiser A, DeCarli C, Au R, Himali JJ, Kelly-Hayes M, et al. Association of mri markers of vascular brain injury with incident stroke, mild cognitive impairment, dementia, and mortality: The framingham offspring study. *Stroke; a journal of cerebral circulation*. 2010;41:600-606

21. Massaro JM, D'Agostino RB, Sr., Sullivan LM, Beiser A, DeCarli C, Au R, et al. Managing and analysing data from a large-scale study on framingham offspring relating brain structure to cognitive function. *Statistics in medicine*. 2004;23:351-367

22. DeCarli C, Miller BL, Swan GE, Reed T, Wolf PA, Garner J, et al. Predictors of brain morphology for the men of the nhlbi twin study. *Stroke; a journal of cerebral circulation*. 1999;30:529-536

23. Kurland BF, Johnson LL, Egleston BL, Diehr PH. Longitudinal data with follow-up truncated by death: Match the analysis method to research aims. *Stat Sci*. 2009;24:211
